# The relationship between sleep problems and suicidality in Bipolar Disorder: a Systematic Review and a Meta-analysis

**DOI:** 10.1192/j.eurpsy.2024.206

**Published:** 2024-08-27

**Authors:** C. Possidente, M. Bort, M. De Prisco, V. Oliva, G. Fico, L. Bracco, C. Sommerhoff, L. Montejo, A. Murru, E. Vieta

**Affiliations:** ^1^Departament de Medicina, Facultat de Medicina i Ciències de la Salut, Universitat de Barcelona; ^2^Bipolar and Depressive Disorders Unit, Hospìtal Clinic de Barcelona, Barcelona, Spain; ^3^Bipolar and Depressive Disorders Unit, University of Bologna, Bologna, Italy; ^4^Institut d’Investigacions Biomèdiques August Pi i Sunyer, Barcelona; ^5^Centro de Investigación Biomédica en Red de Salud Mental (CIBERSAM), Instituto de Salud Carlos III, Madrid, Spain; ^6^Department of Biomedical and Neuromotor Sciences, University of Bologna, Bologna; ^7^Institut d’Investigacions Biomèdiques August Pi i Sunyer; ^8^Departament de Medicina, Facultat de Medicina i Ciències de la Salut, Universitat de Barcelona; ^9^Bipolar and Depressive Disorders Unit, Hospìtal Clinic de Barcelona, Barcelona; ^10^Department of Pathophysiology and Transplantation, University of Milan, Milan, Italy

## Abstract

**Introduction:**

Bipolar disorder (BD) is a multifaceted illness encompassing mood, energy, cognitive and biorhythms alterations. Sleep disturbances are common in prodromic, acute and inter-episodic phases of BD. Suicidality presents a known association with sleep disturbances. However, their interplay in BD remains intricate and not fully elucidated.

**Objectives:**

The aim of the present systematic review (SR) and meta-analysis (MA) is to summarise the available evidence and to provide an estimate of the association between sleep disturbances and suicidality, defined as presence of suicide ideation, behaviour and suicide attempts, in patients with BD.

**Methods:**

We conducted a comprehensive literature search following the Preferred Reporting Items for Systematic Reviews and Meta-Analyses (PRISMA) guidelines across PubMed, PsycINFO, and SCOPUS databases. We included all studies reporting an association between sleep problems and suicidal behaviour in BD patients. No language restriction was imposed. Effect sizes were calculated as odds ratio (OR) for dichotomic variables, standard mean difference (SMD) for continuous outcomes, and Spearman’s coefficient (r) for correlations. Heterogeneity was assessed using the I2 statistic. Global inconsistency was evaluated using the Q statistics with the corresponding p-value.

**Results:**

The initial search yielded 911 unique abstracts, of which 62 underwent full-text screening. Fourteen publications were included, comprising twelve cross-sectional and two longitudinal studies. The total sample consisted of 19,601 subjects diagnosed with BD, of which 51.76% were females and 69.52% had a diagnosis of BD type 1. We found that people with BD and sleep disturbances tend to have higher suicidality, both current (SMD=0.79, 95%CI=0.53, 1.05) and lifetime (OR=1.8; 95%CI=1.41, 2.55), when compared with people with BD and no sleep disturbances. Additionally, patients with BD and a history of suicide attempts tend to have more sleep problems (OR=1.37, 95%CI=1.21, 1.55). Moreover, a positive correlation exists between suicidality and poor sleep quality measured by the Pittsburgh Sleep Quality Index (PSQI) (r= 0.24, 95%CI=0.10, 0.36). No heterogeneity was found, except in the subanalysis of correlation (I2=66.67%, Q p-value=0.01).

**Conclusions:**

Our SRMA outlines a positive relation between sleep disturbances and suicidality in patients with BD. The small number of included studies and the scarcity of longitudinal studies, preventing the inference of a causal relationship, represent the major limitations of this study. Also, studies with objective measures of sleep alterations are currently lacking. The prompt recognition, objective measurement, and treatment of sleep alterations could be crucial in averting or reducing suicidal attempts in BD.

**Disclosure of Interest:**

C. Possidente: None Declared, M. Bort: None Declared, M. De Prisco: None Declared, V. Oliva: None Declared, G. Fico Grant / Research support from: Fellowship from “La Caixa” Foundation (ID 100010434 - fellowship code LCF/BQ/DR21/11880019), L. Bracco: None Declared, C. Sommerhoff: None Declared, L. Montejo: None Declared, A. Murru Grant / Research support from: Spanish Ministry of Science and Innovation (PI19/00672, PI22/00840) integrated into the Plan Nacional de I+D+I and co-financed by the ISCIII-Subdireccion General de Evaluacio ´n and the Fondo Europeo de Desarrollo Regional (FEDER), E. Vieta Grant / Research support from: Spanish Ministry of Science and Innovation (PI18/ 00805, PI21/00787) integrated into the Plan Nacional de I+D+I and cofinanced by the ISCIII-Subdireccio ´n General de Evaluacio ´n and the Fondo Europeo de Desarrollo Regional (FEDER); the Instituto de Salud Carlos III; the CIBER of Mental Health (CIBERSAM); the Secretaria d’Universitats i Recerca del Departament d’Economia i Coneixement (2017 SGR 1365), the CERCA Programme, and the Departament de Salut de la Generalitat de Catalunya for the PERIS grant SLT006/17/00357; the European Union Horizon 2020 research and innovation program (EU.3.1.1. Understanding health, wellbeing and disease: Grant No 754907 and EU.3.1.3. Treating and managing disease: Grant No 945151).

